# Clinical and Functional Outcomes of Posterior Shoulder Fracture-Dislocation: A Retrospective Case Series

**DOI:** 10.7759/cureus.107810

**Published:** 2026-04-27

**Authors:** Parash Bhandari, Kashif Memon, Tayiba Amin, Majid Ali, Tahreem Fatima

**Affiliations:** 1 Trauma and Orthopedics, Queen Elizabeth Hospital Birmingham, Birmingham, GBR

**Keywords:** functional outcome, modified mclaughlin procedure, oxford shoulder instability score, posterior shoulder fracture-dislocation, shoulder instability

## Abstract

Background

Posterior shoulder fracture-dislocation (PSFD) is a rare and often misdiagnosed condition, lacking standardized treatment guidelines. Both conservative and operative approaches are used, but long-term stability outcomes remain unclear. This study evaluates the functional and stability outcomes in patients treated for PSFD using the Oxford Shoulder Instability Score (OSIS) and range of motion (ROM).

Methods

This case series includes 22 patients (26 shoulders) diagnosed with PSFD at University Hospitals Birmingham between January 2020 and December 2024. We retrospectively analyzed patients treated either conservatively or operatively based on fracture severity and surgeon preference. Functional outcomes were assessed at the most recent available follow-up using the OSIS and ROM, including abduction, flexion, and external rotation. Regression analysis was performed to evaluate the relationship between ROM parameters and OSIS using standard software, with statistical significance set at p < 0.05.

Results

A total of 26 shoulders were treated. Nine shoulders (34.6%) underwent closed relocation followed by physiotherapy, while 17 (65.4%) required surgical intervention, including open reduction internal fixation (three shoulders; 11.5%), arthroplasty (five shoulders; 19.2%), and the modified McLaughlin procedure (nine shoulders; 34.6%). Outcomes were assessed using the OSIS and ROM at follow-up visits and through telephone consultations. The average OSIS score was 35.69 (range: 10-48). ROM assessments showed a mean abduction of 91.82° (range: 20-180°), flexion of 100.91° (range: 20-180°), and external rotation of 35.68° (range: 10-60°).

Conclusions

Both conservative and operative treatments led to improved functional outcomes, with the modified McLaughlin procedure showing favorable results in maintaining shoulder stability and function. The OSIS provided valuable insight into recovery. Despite variability in ROM, overall outcomes were satisfactory. These findings support individualized treatment approaches, although further multicentric studies with larger sample sizes are required to refine treatment guidelines.

## Introduction

Posterior shoulder fracture-dislocation (PSFD) is a relatively rare and often underdiagnosed injury, accounting for approximately 2-4% of all shoulder dislocations [1,2]. Unlike anterior shoulder dislocations, posterior dislocations typically result from high-energy trauma, such as motor vehicle accidents, falls, or seizure activity [3]. PSFD is frequently misdiagnosed due to subtle clinical signs and inconclusive radiographs, often leading to confusion with other shoulder conditions [4]. The injury occurs when a force is applied posteriorly to the shoulder, commonly resulting in an associated fracture of the posterior humeral head and/or glenoid rim [5]. This combination of dislocation and fracture complicates management, as it may lead to significant instability, early-onset osteoarthritis, and reduced range of motion (ROM) [6].

The treatment of PSFD can be broadly divided into conservative and operative approaches. Conservative management typically involves reduction and initial immobilization followed by rehabilitation, which aims to restore the status quo and is primarily used for simple, noninvolving fractures [7]. In more complex fracture patterns or in cases of recurrent instability, surgical intervention may be required. Different surgical options have been described, including open reduction and internal fixation (ORIF), shoulder arthroplasty, and the McLaughlin or modified McLaughlin procedures, which aim to provide stability to the glenohumeral joint by transferring the subscapularis tendon to the reverse Hill-Sachs defect [8]. Despite multiple procedures, there is no consensus on the optimal approach in the management of PSFD. Moreover, the post-therapy outcomes of such treatments have not been evaluated in large samples, particularly to assess their long-term stability and gains in functionality.

The Oxford Shoulder Instability Score (OSIS), a validated tool for assessing shoulder stability and function, provides a useful metric for evaluating these outcomes [9]. This study aims to evaluate the clinical and functional outcomes of PSFD using the OSIS and ROM. In addition, the study explores differences between conservative and operative management and examines the relationship between ROM and functional recovery. We hypothesize that operative management, particularly in complex fracture patterns, is associated with improved functional outcomes and that external rotation is positively associated with OSIS.

## Materials and methods

**Figure 1:**
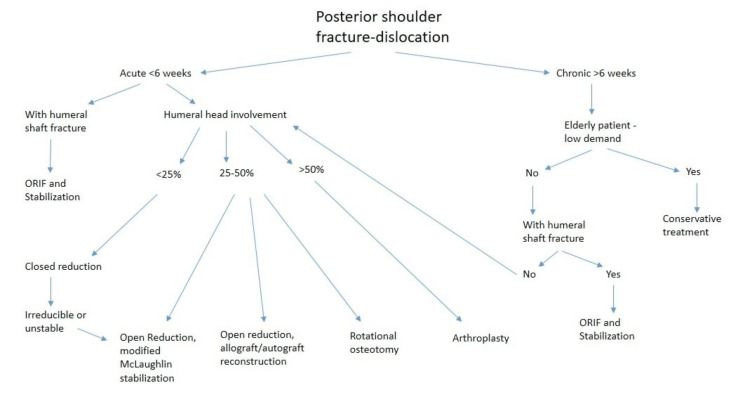
General treatment algorithm for PSFD ORIF, open reduction and internal fixation; PSFD, posterior shoulder fracture-dislocation Adapted from Paparoidamis et al. (2021) [5]

This retrospective descriptive case series with exploratory analysis included 22 patients (26 shoulders) diagnosed with PSFD at University Hospitals Birmingham between January 2020 and December 2024. Patients were selected based on confirmed diagnoses of PSFD, and treatment choices were recorded. To provide context for this study, a general treatment algorithm for PSFD is presented in Figure 1 [5]. Functional outcomes (OSIS and ROM) were assessed at the most recent available follow-up. Due to the retrospective design, follow-up duration varied between patients.

Treatment allocation was based on fracture characteristics, degree of instability, and the surgeon’s clinical judgment. Conservative management was generally used for minimally displaced fractures without persistent instability, while operative management was performed in more complex fracture patterns or where stable reduction could not be achieved. No standardized classification system was used, which reflects real-world practice but introduces potential selection bias.

All patients aged 17 years or older who were diagnosed with a PSFD confirmed on radiographic imaging and who underwent either conservative or operative treatment were included in the study. Patients were excluded if they were younger than 17 years, had an active shoulder infection, or had a known malignancy involving the shoulder.

Patient records were retrospectively reviewed using multiple institutional sources, including the Patient Information and Communication System, the Picture Archiving and Communication System, the clinical portal, the Galaxy theater system, the orthopedic trauma database, and upper limb multidisciplinary team review notes. Relevant demographic, clinical, radiographic, treatment, and follow-up data were obtained from these sources for analysis.

Patients were managed either conservatively or operatively. Conservative treatment consisted of closed reduction followed by a structured physiotherapy program (nine shoulders; 34.6%). Operative management was undertaken when anatomical restoration was not achievable and included ORIF (three shoulders; 11.5%), shoulder arthroplasty (five shoulders; 19.2%), or the Modified McLaughlin procedure (nine shoulders; 34.6%).

Outcome measures included both patient-reported and clinical assessments. The OSIS was documented following treatment during routine follow-up visits and, when necessary, through telephone consultations [9]. ROM was evaluated clinically through visual estimation and documented in degrees of abduction, flexion, and external rotation based on standardized anatomical positions and reference values established by the American Academy of Orthopaedic Surgeons [10]. Visual estimation was utilized due to the retrospective nature of the study, a method previously validated for its clinical reproducibility in shoulder assessment [11].

Regression analysis was used to evaluate the relationship between the OSIS and ROM. Statistical analyses were performed using standard software, with significance set at p < 0.05. Given the small sample size, regression analysis was performed as an exploratory assessment of associations and should be interpreted with caution.

## Results

The study included 22 patients (n = 22) with a total of 26 affected shoulders. The mean age of the patients was 49.9 ± 15.0 years (range: 28-75), comprising 19 (86.4%) males and three (13.6%) females. The clinical and demographic characteristics of the study population are summarized in Table 1.

**Table 1:** Clinical and demographic characteristics of patients with PSFD ER, external rotation; GT, greater tuberosity; LT, lesser tuberosity; ORIF, open reduction and internal fixation; OSIS, Oxford Shoulder Instability Score; PSFD, posterior shoulder fracture-dislocation; TSA, total shoulder arthroplasty

Serial no.	Side	Age/sex	Mechanism of injury	Diagnosis	Treatment	OSIS	Abduction	Flexion	ER (arm at side)
1	Right	55/Male	Seizure	Reverse Hill-Sachs lesion	Modified McLaughlin	30	40	100	40
	Left	55/Male	Seizure	Reverse Hill-Sachs lesion	Modified McLaughlin	30	40	100	40
2	Left	63/Male	Mechanical fall	Fracture-dislocation (surgical neck + proximal diaphysis)	ORIF	41	150	120	60
3	Right	50/Male	Mechanical fall	Large reverse Hill-Sachs + GT + LT fracture	ORIF + allograft	33	150	130	60
4	Left	70/Female	Mechanical fall	Comminuted intra-articular fracture-dislocation	Reverse TSA	44	90	90	50
5	Left	55/Male	Unknown	Large, engaging reverse Hill-Sachs + surgical neck fracture	Hemiarthroplasty	15	40	50	15
6	Right	38/Male	Assault and fall	Reverse Hill-Sachs lesion	Conservative	35	50	60	25
7	Left	74/Male	Mechanical fall	Comminuted humeral head fracture	Reverse TSA	48	110	110	60
8	Left	27/Male	Seizure	Reverse Hill-Sachs + GT + LT fracture	Modified McLaughlin	28	180	180	60
9	Right	46/Male	Seizure	Reverse Hill-Sachs?	Modified McLaughlin	13	20	20	10
10	Right	75/Female	Seizure	Large reverse Hill-Sachs lesion	Conservative	38	90	90	20
	Left	75/Female	Seizure	Fracture-dislocation	Conservative	41	90	90	25
11	Right	39/Male	Mechanical fall	Small reverse Hill-Sachs lesion	Conservative	43	160	120	50
12	Right	28/Female	Seizure	Fracture-dislocation	Conservative	48	180	180	60
	Left	28/Female	Seizure	Fracture-dislocation	Conservative	39	110	100	35
13	Right	32/Male	While turning in bed	Shallow reverse Hill-Sachs	Conservative	43	100	100	45
14	Right	32/Male	Road traffic accident	Small reverse Hill-Sachs lesion	Conservative	43	160	160	50
15	Right	59/Male	Assault	Large anterior humeral head defect	Modified McLaughlin	40	90	90	30
16	Left	40/Male	Assault	Large engaged reverse Hill-Sachs	Conservative	37	80	90	20
17	Right	74/Male	Road traffic accident	Comminuted surgical neck + intra-articular extension	Reverse TSA	43	90	90	35
18	Right	56/Male	Road traffic accident	Comminuted humeral head + GT + LT fracture	Hemiarthroplasty	15	40	30	10
19	Left	34/Male	Seizure	Large reverse Hill-Sachs + GT + LT fracture	Modified McLaughlin	27	45	102	48
20	Right	59/Male	Mechanical fall	Proximal diaphyseal fracture	ORIF	10	90	90	20
21	Right	41/Male	Seizure	Multifragmentary reverse Hill-Sachs	Modified McLaughlin	45	50	90	30
	Left	41/Male	Seizure	Multifragmentary reverse Hill-Sachs	Modified McLaughlin	45	60	90	20
22	Left	40/Male	Seizure	Fracture anterior humeral head + anatomical neck + LT	Modified McLaughlin	34	90	90	30

Functional outcomes, OSIS, and ROM were assessed at the shoulder level (n = 26). The mean OSIS across all patients was 35.69, with a range of 10-48. Shoulder ROM was assessed in abduction, flexion, and external rotation. The mean abduction was 91.82° (range, 20-180°), the mean flexion was 100.91° (range: 20-180°), and the mean external rotation was 35.68° (range: 10-60°). These data are summarized in Table 2.

**Table 2:** OSIS and shoulder ROM in study patients OSIS, Oxford Shoulder Instability Score; ROM, range of motion

Outcome measures	Mean (n = 26)	Range
Average OSIS	35.69	10-48
ROM (°)		
Abduction	91.82	20-180
Flexion	100.91	20-180
External rotation	35.68	10-60

To assess the relationship between functional outcome and ROM, a multiple regression analysis was conducted using the OSIS score as the dependent variable and ROM measures (abduction, flexion, and external rotation) as predictors. The regression model was statistically significant (F (3, 22) = 4.554, p = 0.0144) and explained approximately 41.8% of the variance in OSIS scores (R² = 0.418, adjusted R² = 0.326). The multiple regression and the regression model summary are provided in Table 3 and Table 4, respectively.

**Table 3:** Multiple regression summary ROM, range of motion

Variable	Coefficient	SE	t-value	p-value
Intercept	2.360	3.892	0.606	0.551
Abduction ROM	-0.25	0.04	2.774	0.093
Flexion ROM	0.26	0.047	1.896	0.097
External Rotation ROM	0.46	0.076	1.981	0.060

**Table 4:** Regression model summary

Metric	Value
R²	0.418
Adjusted R²	0.326
F-statistic	4.554
p-value (model)	0.0144

The regression equation was:

$\text{OSIS score} = 2.36 + 0.11 \times (\text{abduction ROM}) + 0.09 \times (\text{flexion ROM}) + 0.15 \times (\text{external rotation ROM})$

Among the predictors, external rotation ROM demonstrated the strongest positive trend with OSIS score; however, this did not reach conventional statistical significance and should be interpreted as an exploratory finding. Abduction ROM showed a negative association, while flexion ROM showed a modest positive trend; however, all p-values were marginal (external rotation = 0.060, abduction p = 0.093, flexion p = 0.097).

**Figure 2:**
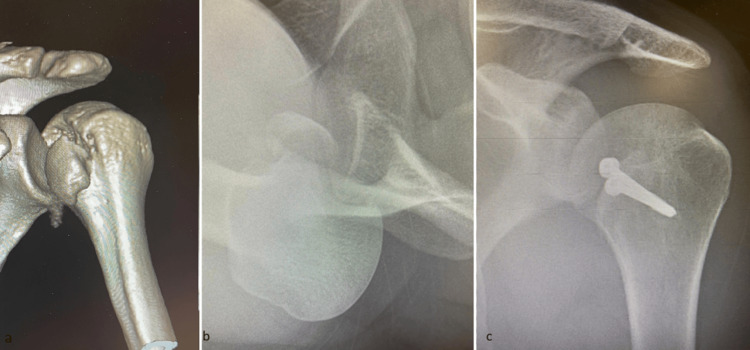
PSFD managed with the modified McLaughlin procedure (a) 3D CT reconstruction showing a posterior fracture-dislocation with reverse Hill-Sachs defect. (b) Preoperative axillary radiograph demonstrating a locked PSFD with associated humeral head impaction. (c) Postoperative radiograph showing restoration of joint congruency following the modified McLaughlin procedure. PSFD, posterior shoulder fracture-dislocation

**Figure 3:**
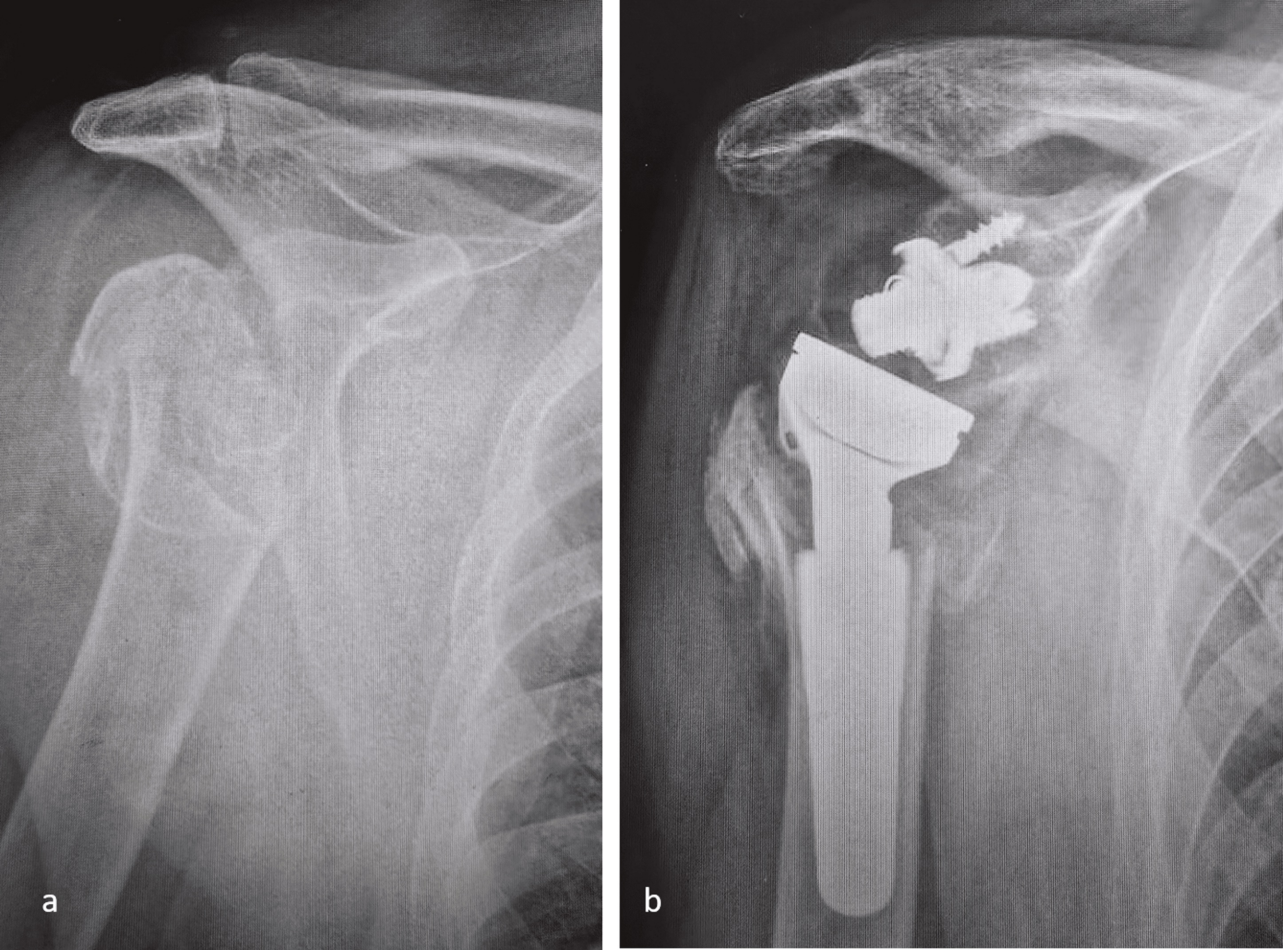
Comminuted proximal humerus fracture managed with reverse TSA (a) Preoperative radiograph demonstrating a severely comminuted humeral head fracture associated with posterior instability. (b) Postoperative radiograph showing a reverse TSA performed for reconstruction and stability. TSA, total shoulder arthroplasty

**Figure 4:**
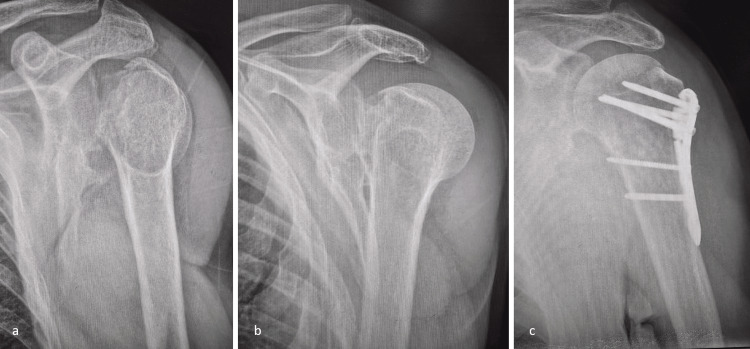
PSFD treated with ORIF (a, b) Initial radiographs demonstrating posterior dislocation with a displaced proximal humerus fracture. (c) Postoperative radiograph illustrating anatomic reduction and stable internal fixation. ORIF, open reduction and internal fixation; PSFD, posterior shoulder fracture-dislocation

Representative radiographic examples of the fracture patterns and treatments used in this cohort are shown in Figure 2, Figure 3, and Figure 4. Figure 2 demonstrates a posterior fracture-dislocation managed with the modified McLaughlin procedure; Figure 3 illustrates a comminuted humeral head fracture treated with reverse total shoulder arthroplasty (TSA); and Figure 4 shows a posterior fracture-dislocation managed with ORIF.

## Discussion

This study aimed to document the outcomes of conservative and operative management of PSFDs, focusing on functional recovery, stability, and ROM. The results indicate that both treatment approaches improve shoulder functionality; however, operative management demonstrated a trend toward improved outcomes in more complex cases in terms of shoulder strength and mobility. These findings are consistent with previously published studies, which suggest that surgical treatment, whether ORIF, the modified McLaughlin procedure, or shoulder arthroplasty, provides better long-term outcomes for complex shoulder fractures [12,13].

The improved outcomes in the operative group can be attributed to surgical restoration of anatomical alignment, reestablishment of joint stability, and prevention of recurrent dislocations. ORIF, performed in three cases, allowed for anatomical restoration of complex proximal humerus fractures, improving joint congruity, mobility, and strength, consistent with previously reported results [14]. The modified McLaughlin procedure, performed in 9 cases for posterior fracture-dislocations with reverse Hill-Sachs lesions, provided excellent stability and functional recovery, as supported by recent clinical evidence showing significant improvements in functional outcomes [15]. In contrast, conservative treatment achieves satisfactory long-term stability primarily in less complex fractures and in patients without recurrent instability, highlighting its limited applicability in more severe cases. Moreover, the operative group demonstrated greater ROM values, although formal statistical comparison between groups was not performed, consistent with previous studies indicating that surgical intervention can enhance joint mobility.

A recent systematic review and meta-analysis conducted by Crowley et al. demonstrated the effectiveness of TSA over hemiarthroplasty in patients with locked posterior dislocations, and, notably, better TSA outcomes were found to be significantly associated with advanced patient age, validating our finding that elderly patients benefit maximally from joint reconstruction procedures [16].

Kabelka et al. also reported their experience in managing posterior shoulder dislocation and humeral head compression fracture using the McLaughlin technique and its modification, and they concluded that their results were excellent and that patient-tailored surgical methods were important in managing complex shoulder dislocation [17].

A systematic review by Berk et al. assessed the clinical and radiographic follow-up after the modified McLaughlin approach in the treatment of locked posterior shoulder dislocations, emphasizing excellent functional outcomes and a low complication rate, which further supports individualized surgery in the treatment of posterior glenohumeral dislocations [18].

The OSIS was sensitive in assessing stability and functional outcomes across the treatment group. Both the operative and conservative cohorts demonstrated notable improvement in OSIS, indicating that even patients managed conservatively can achieve good shoulder function with adequate rehabilitation. Nevertheless, the operative group showed greater improvement, with higher OSIS reflecting superior shoulder stability and function. These results support the OSIS as a practical and reliable outcome measure for shoulder instability in clinical practice [9].

Regression analysis provided further insight into the relationship between specific ROM components and functional outcome. External rotation ROM proved to be the strongest predictor of OSIS, emphasizing the importance of incorporating targeted external rotation exercises into rehabilitation programs. Interestingly, abduction ROM demonstrated a negative coefficient, likely reflecting pain or compensatory movements at full abduction. These findings align with clinical observations and support the development of specialized rehabilitation strategies.

This study has several limitations. It is a retrospective case series with a relatively small sample size, limiting generalizability. Treatment allocation was not standardized and was influenced by surgeon judgment and fracture characteristics, introducing potential selection bias. Outcomes were analyzed as pooled data without formal subgroup comparison between treatment modalities, limiting the ability to draw definitive conclusions regarding treatment superiority. ROM was assessed through visual estimation and may be subject to interobserver variability. Additionally, regression analysis was performed on a small sample and should be interpreted as exploratory. Future prospective multicenter studies with standardized protocols are required.

## Conclusions

The management of PSFDs should be individualized based on fracture characteristics and patient factors. Both conservative and operative approaches resulted in satisfactory functional outcomes in this cohort. Operative management showed favorable trends in more complex cases, particularly with the modified McLaughlin procedure; however, these findings are descriptive and require further validation. External rotation may play an important role in functional recovery, although this association remains exploratory. Larger prospective studies are required to establish definitive treatment guidelines.
